# Phenotypic and functional characteristics of murine CD11c+ B cells which is suppressed by metformin

**DOI:** 10.3389/fimmu.2023.1241531

**Published:** 2023-09-06

**Authors:** Ivan Ramirez De Oleo, Vera Kim, Yemil Atisha-Fregoso, Andrew J. Shih, Kyungwoo Lee, Betty Diamond, Sun Jung Kim

**Affiliations:** ^1^ Center for Autoimmune, Musculoskeletal and Hematopoietic Diseases, The Feinstein Institutes for Medical Research, Manhasset, NY, United States; ^2^ Center for Genomics and Human Genetics, The Feinstein Institutes for Medical Research, Manhasset, NY, United States; ^3^ Department of Biology at Hofstra University, Hempstead, NY, United States; ^4^ Department of Molecular Medicine, Donald and Barbara Zucker School of Medicine at Hofstra University/Northwell, Hempstead, NY, United States

**Keywords:** follicular T helper cells (Tfh), systemic lupus erythematosus (SLE), age-associated B cells (ABCs), mouse model, metabolism, autoimmune diseases

## Abstract

Since the description of age-associated or autoimmune-associated B cells (ABCs), there has been a growing interest in the role of these cells in autoimmunity. ABCs are differently defined depending on the research group and are heterogenous subsets. Here, we sought to characterize ABCs in Sle1/2/3 triple congenic (TC) mice, which is a well accepted mouse model of lupus. Compared to follicular (FO) B cells, ABCs have many distinct functional properties, including antigen presentation. They express key costimulatory molecules for T cell activation and a distinct profile of cytokines. Moreover, they exhibit an increased capacity for antigen uptake. ABCs were also compared with germinal center (GC) B cells, which are antigen activated B cell population. There are several phenotypic similarities between ABCs and GC B cells, but GC B cells do not produce proinflammatory cytokines or take up antigen. While T cell proliferation and activation is induced by both FO B and ABCs in an antigen-dependent manner, ABCs induce stronger T cell receptor signaling in naïve CD4+ T cells and preferentially induce differentiation of T follicular helper (Tfh) cells. We found that ABCs exhibit a distinct transcriptomic profile which is focused on metabolism, cytokine signaling and antigen uptake and processing. ABCs exhibit an increase in both glycolysis and oxidative phosphorylation compared to FO B cells. Treatment of ABCs with metformin suppresses antigen presentation by decreasing antigen uptake, resulting in decreased Tfh differentiation. Taken together, these findings define a fundamental connection between metabolism and function within ABCs.

## Introduction

B cells play critical roles in host defense through their capacity to produce antibodies and cytokines as well as present antigens to T cells. Uncontrolled B cell activation or reactivity to self-antigens contributes to deleterious autoimmune responses. In addition to conventional B2 and B1 cells, a distinct and unusual B cell subset has been identified in humans and mice. It is characterized by expression of the integrin, CD11c, and T-box transcription factor (T-bet) and lack of the follicle-homing chemokine receptor (CXCR5) and CD21 (1, 2). They are termed age-associated or autoimmune-associated B cells (ABCs). Initial studies characterized ABCs by expression of CD11c (alone or with CD11b) in aged mice, and in multiple mouse models for systemic lupus erythematosus (SLE) expanded (2–4). Similarly, ABCs are expanded in a mouse model of rheumatoid arthritis (5). There is an acute increase of ABCs induced by viral infection in non-autoimmune mice (6). In a similar manner, there are numerous examples of ABCs in the context of autoimmune disease in humans. While present at low frequency in the peripheral blood of healthy individuals, ABCs are expanded in aged individuals (especially females), in patients with chronic viral infection, and in individuals with several autoimmune diseases including Systemic Lupus Erythematosus (SLE), Rheumatoid Arthritis (RA), and multiple sclerosis (MS) and in common variable immunodeficiency (CVID) [review in (7)].

Transcriptional profiling of ABCs suggests that they have a memory-like phenotypes, such as increased proportion of IgD-/IgM-, and high expression of activation markers such as CD80 and CD86 (2). They also express high levels of CXCR6 and CXCR3, which are involved in homing to sites of inflammation (8, 9). Studies from SLE patients suggest ABCs are precursors of autoantibody-producing plasma cells. IgD-/CD27-, double-negative 2 (DN2) or CD11c+T-bet+ B cells can differentiate into Ig-secreting plasma cells in ex vivo stimulation conditions that include stimulation with a TLR7 agonist or IL-21 (1, 10). Murine CD11c+ B cells, which were identified by the Marrack and Cancro laboratories, have many similarities to CD11c+ atypical memory B cells in humans (2, 11);. Like human ABCs, murine CD11c+ B cells express T-bet and activation markers. ABCs are hyporesponsive to stimulation through the B cell receptor (BCR) and CD40 but are responsive to toll-like receptor (TLR) 7/9 stimulation and produce autoantibodies and cytokines, such as IL-4 and IL-10 (11). In the acute LCMV infection model, T-bet+CD11c+ ABCs can produce high amounts of anti-viral antibody following ex vivo stimulation with a TLR7 agonist (12). In that study, most ABCs developed independent of a germinal center (GC) response. In autoimmune mice, ABCs produce more antichromatin IgG2a than non-ABCs (3). The importance of TLR7 signaling in the production of autoantibodies in lupus-prone mice is demonstrated by the autoantibody production that occurs with TLR7 inhibition (13). ABCs can form stable interaction with T cells and act as antigen presenting cells (APCs) (14). They reside at the T/B border in the spleen which increases interactions with T cells. Their localization is partly due to an increased expression of CCR7 and decreased expression of CXCR5. It has been shown that ABCs contribute to the aberrant differentiation of T follicular helper (Tfh) cells leading to dysregulation of an antigen-specific GC response (15). The pathogenic role of CD11c+ ABCs in autoimmune disease is also evident from studies showing that the removal of these cells significantly ameliorates disease severity and disease progression in the SHIP-deficient B cell mouse model of lupus (15).

Distinct metabolic pathways are required for the specific functions of cells, and modulation of cellular metabolism may normalize cell-specific pathogenic function in disease conditions. Here, we investigated the cellular and molecular mechanisms of ABCs as APCs in Sle1/2/3 triple congenic (TC), a mouse model of SLE. Transcriptome analyses suggested that ABCs have increased expression of molecules involved in antigen uptake, cytokine signaling, and metabolism. ABCs exhibit increased expression of activation markers and a distinct cytokine profile. They express several receptors which are involved in phagocytosis and they uptake a higher amount of soluble antigen than non-ABCs. ABCs induce strong T cell activation and differentiation skewed toward IL-21 producing Tfh cells. They display more active mitochondrial metabolism and reactive oxygen species (ROS) production than FO B cells. Blocking mitochondrial metabolism and mitochondrial ROS production by metformin prevents their ability to induce Tfh differentiation, in part through an inhibition of antigen uptake.

## Materials and methods

### Mice

C57BL/6J, B6; NZM-*Sle1^NZM2410/Aeg^ Sle2^NZM2410/Aeg^ Sle3^NZM2410/Aeg^*/LmoJ (stock number 007228) triple congenic (TC) mice, B6.lpr (stock number 000482) (16), C57BL/6J (stock number 000664) and C57BL/6-Tg(Nr4a1-EGFP/cre) (Nur77-GFP, stock number 016617) (17) were purchased from the Jackson Laboratory (Bar Harbor, ME) and bred in house. OT II. Nur77-GFP mice were generated by breeding Nur77-GFP and OT II (B6.Cg-Tg (TcraTcrb425Cbn/J, stock number 004194) and maintained at the FIMR. Female mice were used for all experiments. ABCs and FO B cells were isolated and characterized from 8–10-month-old mice, and 6-8 weeks old mice were used to isolate naïve T cells. We confirmed the disease status by measuring anti-dsDNA IgG and proteinuria. TC and B6.lpr mice were positive for both anti-dsDNA IgG and proteinuria, compared with age matched C57BL/6J mice. Mice were house at the center for Comparative Medicine at the FIMR with standard 12 hours light/darkness cycle, ambient temperature 23 °C and were provided rodent diet (Lab Diet, St. Louis, MO) and water ad libitum.

### Metformin treatment and immunization

To examine the effect of metformin on ABCs in TC mice, metformin (2.5 mg per mouse) or saline was administrated i.p. daily for 1 week. To induce GC B cells, C57BL/6J mice were immunized with NP-CGG (100 µg) in alum intraperitoneally.

### Phenotyping and isolation of cells by flow cytometry

Single cell suspension was prepared from the spleen using a stand protocol. After removing red blood cells (RBC) by RBC lysis buffer (ThermoFisher Scientific, cat no. 00-4333-57, Waltham, MA), the cell suspension was filtered through 70 µm filter. Cell counts were obtained on Cellometer auto 2000 cell counter (Nexcelome, Lawrence, MA) using acridine orange to discriminate nucleated cells from debris and propidium iodide to discriminate dead cells. For immune cell phenotyping, live cells were identified by exclusion of live/dead cell dye (fixable viability dye eFluor506, Invitrogen) positive cells, and were stained on EasySep buffer [HBSS 1x supplemented with 0.5 M EDTA and 2% heat-inactivated fetal bovine serum (FBS)]. For surface staining, single cell suspension was prepared and washed with EasySep buffer. 1-5 x 10^6^ cells were incubated with antibody cocktail for 20 min on ice in dark and washed with 10 volumes of EasySep buffer three times. Intracellular staining was performed with True Nuclear staining kit (Biolegend) followed manufacture’s protocol. After completion of surface staining, cells were fixed with fixation solution for 45 min at room temperature in dark. Fixation solution was washed with 1x Perm solution twice, and antibodies for intracellular staining (in Perm solution) was incubated for 45 min at room temperature in dark. Flow cytometry was performed on BD FACS Symphony A3 (BD Biosciences) for analysis or BD FACSAria IIu (BD Biosciences) for sorting.

Antibodies were purchased from company and company name and clone number are indicated as followed: anti-CD11c (BD Pharmingen, clone# N418), anti-CD93 (eBioscience, clone# AA4.1), anti-CD95 (BD Bioscience, clone#Jo2), anti-GL7 (eBioscience, clone#GL-7), CD21/CD35 (Biolegend, clone#7E9), anti-B220 (BD Bioscience, clone# RA3-6B2), anti-CD19 (BD Pharmingen, clone# 1D3), anti-CD23 (Biolegend, clone# B3B4), purified rat anti-mouse CXCR5 (BD Pharmingen, cat# 551961), goat-anti-rat IgG (H+L)-Biotin (Southern biotech, cat#3030-08), anti-TCRβ (Biolegend, clone# H57-597), anti-CD4 (Biolegend, clone# GK1.5), anti-PD-1 (BD Bioscience, clone# J43), anti-CD44 (BD Bioscience, clone# IM7), anti-CD62L (BD Bioscience, clone# MEL-14), anti-CD11b (Biolegend, clone# M1/70), anti-MHC II (eBioscience, clone# M5/114.15.2), anti-CD36 (eBioscience, clone# HM36), anti-CD86 (eBioscience, clone# GL1), anti-SLAM (Biolegend, clone# TC15-12F12.2), anti-ICOS-L(eBioscience, clone# HK5.3), anti-IgD (BD Bioscience, clone# 11-26c-2a), anti-IgM (eBioscience, cat# 11/41), anti-T-Bet (Biolegend, clone# 4B10), anti-IgG1 (BD Pharmingen, clone# A85-1), anti-IgG2a/2b (BD Pharmingen, clone# R2-40), anti-IgG3 (BD Pharmingen, clone# R40-82), anti-IgA (eBioscience, clone# mA-6E1), anti-Ki67 (BD Bioscience, clone# B56), anti-BCL6 (eBioscience, clone# BCL-DWN), anti-IL-17 (eBioscience, clone# eBio1787), anti-IFNγ (eBioscience, clone#XMG1.2), and anti-IL-4 (Biolegend, clone# 11B11). Intracellular IL-21 was detected by IL-21 R-Fc (R&D System) followed by AF488-cojugated goat anti-human IgG (Jackson Laboratory).

### Quantification of OVA uptake by flow cytometry and Image stream

ABCs or FO B cells were incubated alone or with 10 µg/ml FITC-OVA (Invitrogen) or DQ-OVA (Invitrogen) for 30 min at 37°C for internalization. OVA uptake by B cell subsets was measured by incubation of splenocytes with 10 µg/ml of OVA-AF488 or OVA PE-TR (both were purchased from Invitrogen) for 30 min at 37 °C. After incubation, free OVA was removed by washing with ice-cold staining buffer and B cell subsets were identified by staining for surface markers. To block the CD36, 10 µg/ml of control IgA (Abcam, S107, ab37322) or anti-CD36 neutralizing antibodies (Cayman, clone: JC63.1) were treated with B cells for 2 hours before OVA incubation. After the incubation, cells were washed with ice-cold staining buffer three times and resuspend in PBS at 10^6^/ml concentration. Percent of OVA positive B cells and intensity of OVA in B cells were quantified by flow cytometry analysis. Internalized OVA was identified by Imaging flow cytometry. Imaging flow cytometry was performed on an ImageStreamX Mark II. 1000 of CD19+ single live B cells were acquired for the analysis in each sample.

### *In vitro* differentiation of Tfh by B cells and metformin treatment

ABCs and FO B cells were purified from spleens by cell sorter and plated into round bottom 96 well plate (10^5^ cells/well). OVA protein (EndoFit OVA was purchased from InvivoGen, cat# vac-pova) was added (10 µg/ml) for 3 hours before addition of T cells. Naïve CD4 T cells were isolated from spleens of OT II mice by CD4+ naïve T cell isolation kit from Stem cell technologies (cat# 19765A, Stem cell technology). Purity of naïve CD4+ T cells was confirmed by flow cytometry and average 95% purity was obtained. Isolated naïve CD4+ T cells were added to the B cell culture at the ratio of 2:1 (B cells: T cells) in total 200 µl. Cells were incubated at 37 °C, CO_2_ (5%) incubator for 3-4 days. For metformin inhibition assay, isolated B cells were incubated with 1 mM metformin (item no. 13118, Cayman chemical) or phenformin (item no. 14997, Cayman chemical) for 2-3 hours and cells were washed with media 3 times to remove free metformin in the following culture.

### Cytokine measurement

Secreted cytokines from B cells were measured by either mouse proinflammatory panel 1 from mesoscale discovery (MSD, cat# N05048A-1) followed by manufacture’s protocol. All the samples were measured in duplicates, and any value within the linear range of each cytokine was taken as data.

Intracellular cytokine production was measured by flow cytometry. Briefly, cells were stimulated with PMA (100 ng/ml) and ionomycin (1 µg/ml) for 6 hours on the day of assay. To accumulate the cytokine inside of cells, BFA (20 µg/ml) was added during the last 4 hours of stimulation. Staining procedure for intracellular staining and surface staining are followed as described in previous section. Frequency of each cytokine positive cells was calculated after exclusion of dead cells.

### Lactate/ATP measurement

B cells were isolated and cultured overnight. Next day, intracellular ATP level was measured from cell pellet by Luminescent ATP measure kit (Abcam) and culture supernatant was collected for lactate assay by Lactate colorimetric/fluorometric assay kit followed by manufacture’s protocol (BioVision). Luminescence of ATP assay was measured by Victor 1420 (Perkin Elmer) and lactate assay absorbance (OD570 nm) was measured by Synergy H1 microplate reader (BioTek).

### Intracellular metabolism assay

For mitochondria contents and membrane potential, cells were incubated with MitoTracker red (Invitrogen, cat# M22425) and MitoTracker green (Invitrogen, cat# M7514) at a concentration of 10 nM in complete media for 15 min at 37°C. To measure of glucose uptake, cells were incubated with 100 µM 2-NBDG (Cayman, cat# 11046) for 20 min at 37°C in complete media. Cellular ROS was measured by MitoSOX (1 µM) or H2DCFDA (1 µM). Cells were incubated in 500 µl of MitoSOX or H2DCFDA for 10 min at 37°C. After the labeling, cells were washed three times with staining buffer and analyzed by flow cytometry.

### Seahorse experiments

A Seahorse XFp Extracellular Flux Analyzer was used to measure OCR and ECAR of ABC and FO B cells using the MitoStress test condition by Agilent seahorse. ABC and FO B cells where immobilized to create a monolayer of cells (2 x 10^5^) on XFp microplates using the manufacturer protocol for immobilization of non-adherent cell using Cell-Tak (Corning). Assay medium was made by using XFp Base medium enriched with 2.5 M of Glucose, 100 mM of pyruvate and 200 mM Glutamine per 10 mL of XFp base Medium all from Agilent seahorse. Baseline and maximal ECAR and OCR values were averaged between 3 replicates per sample for 3 successive time intervals.

### RNA purification and RT-qPCR

Total RNA was purified from sorted cells by Direct-zol RNA MicroPrep (ZymoResearch). Genomic DNA was removed by in-column DNA digestion during the RNA preparation. 200 ng of isolated RNA was converted to cDNA by iScript DNA synthesis kit (BioRAD) and quantitative PCR was performed as previously described (18). Primers were purchased from Thermofisher as follows: *Polr2a* (Mm00839503_m1), *Il12a* (Mm00434169_m1), *Il1b* (Mm00434228_m1), *Tnf* (Mm00443258_m1), *Il6* (Mm00446190_m1), *Il10* (Mm01288386_m1), *Hk2* (Mm00443385_m1), *Mthfd2* (Mm00485276_m1), *Slc2a1* (Mm00441480_m1), *Stat5* (Mm03053818_s1), *Cd36* (Mm00432403_m1), *Axl* (Mm00437221_m1), *Hifa* (Mm00468869_m1), *Slc22a1* (Mm00456303_m1), *Slc22a2* (Mm00457295_m1) and *Slc22a3* (Mm00488294_m1). The reference gene, *Polr2a*, was used for normalization and relative expression was calculated by 2^-ΔCt^ method.

### Quantification of mitochondria

To isolate mitochondria DNA and genomic DNA, isolated B cells were lysed, and total DNA was isolated by DNeasy gDNA isolation kit (QIAGEN). In-column Rnase treatment was performed to remove RNA contamination. Mitochondrial DNA was quantified by levels of *Nd1* (NADH dehydrogenase subunit 1) and *Rnr2* (16S ribosomal RNA) and nuclear DNA was quantified by level of actin. Primers for qPCR were purchased from Taqman (Invitrogen); Mm04260181_s1 (*Rnr2*), Mm04225274_s1 (*Nd1*), and Mm00607939_s1 (*Actb*). To determine the mitochondrial DNA contents, relative to nuclear DNA calculated using the following equations: a) ΔC_T_ = (nucDNA C_T_ – mtDNA C_T_), b) Relative mitochondrial DNA content = 2 x 2 ^ΔCT^.

### Quantitation of oxidized lipid

Oxidized lipid was detected by labeling of C11-BODIPY (581/591) (catalog number D3861, TermoFisher), an oxidation-sensitive fluorescent probe (19). Isolated B cells were incubated with 5 µM of C11 for 30 min at 37°C. Labeled cells were washed with washing buffer three times and fluorescence was analyzed by flow cytometry.

### RNA-Seq and bioinformatics

CD11c+ ABCs and FO B cells were freshly isolated and snap-frozen for further process. RNA purification, library preparation and sequencing were performed by Azenta (AZENTA life sciences). Briefly, total RNA was purified, and mRNA was selected with polyA selection. QC of purified RNA and amplified cDNA were confirmed, and only high quality of samples were used for the sequencing. 2x150 base pair, single index sequencing (paired-end) was performed using an Illumina HiSeq sequencer.

QC for fastq files was performed with FastQC. Reads were mapped to the mouse genome (GRCm39) using STAR2 (20). The aligned reads were quantified with htseq-counts to the M29 annotation. Raw counts were compiled to identify differentially expressed gene and analyzed using DESeq2. Genes were considered differentially expressed if they showed at least 1 absolute log2-fold changes and a false discovery rate of <0.05. Pathway/gene set enrichment was done using fGSEA on MSigDB gene sets (21–23). RNA-Seq data was also analyzed by using the RNA-Seq workflow in Petek Flow software (Partek Inc., St. Lousie, MO). Statistical analysis and plotting were performed with R+tidyverse and pheatmap used to draw heatmaps.

### Mass spectrometry-based metabolomic analysis

Sample preparation, process and analysis was followed by previous report with modification (24). 5 x 10^5^ ABCs or FO B cells were sorted by cell sorter. Isolated cells were washed with ice-cold HBSS three times and snap-frozen until following steps. Cells were lysed by incubation with extraction solvents (50:50 v/v methanol:acetonitrile) for 1 hour at -20°C. Lysed cell extracts were centrifuged and upper layer (containing metabolites) were collected and further processed by LC-tandem mass spectrometry (MS/MS). Chromatrography was performed with a Shimadzu Nexera system (Shimadzu, Columbia, MD) coupled with a high-resolution hybrid quadrupole time-of-flight mass spectrometer (TripleTOF 5600, Framingham, MA).

Targeted metabolomic data were processed using Peak View 2.1 and MultiQuanta software version 3.0.2 (SCIEX). Chromatographic peaks of targeted metabolites were annotated, and each identified metabolite was quantified by integrating peak area using MultiQuant software. The quantitative analysis was based on the total peak areas of extracted ion chromatograms of feature ions. Principal component analysis plot and heat map were generated with MetaboAnalyst (v4.1).

### Statistics

Statistical analyses and significance were performed using GraphPad Prism v.9.02 software (GraphPad Software Inc.). Nonparametric student-test, either Mann-Whitney test or Wilcoxon matched-pair test, or one-way ANOVA with Sidak’s correction for multiple comparisons. All the data are reported as mean.

### Study approval

All animal experiments and procedures were performed according to protocols approved by the Institutional Animal Care and Use committee at the Feinstein Institutes for Medical Research (FIMR) (approved protocol no. 2022-022 term I).

## Results

### CD11c+ ABCs are T-bet positive and exhibit features of professional antigen presenting cells

**Figure 1:**
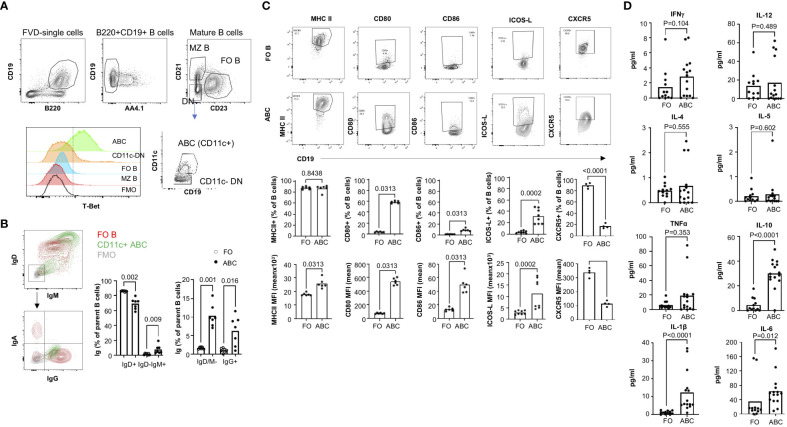
ABCs exhibit increased expression of costimulatory molecules and cytokines. **(A)** gating strategy for the identification of ABCs. ABCs were identified as viable, CD19+B220+AA4.1-CD21-CD23-CD11c+. T-Bet expression was measured in each B cell subset. **(B)** Surface Ig expression of ABCs was assessed by flow cytometry. A representative flow image for Ig expression was made for FO B cells (in red contour), ABCs (in green contour), and FMO control (in grey contour). The percentage of surface Ig expressing B cells from ABCs or FO B cells was calculated (n=3). **(C)** Expression of costimulatory molecules was assessed in ABCs or FO B cells by flow cytometry. Representative flow image is presented on the top row and the percentage of positive or mean fluorescent intensity (MFI) of each molecule is graphed (on the bottom row). (n=3). **(D)** ABCs and FO B cells were purified, and 10^5^ cells were cultured overnight. Cytokine in the supernatant was measured by MSD (n=4). Each dot represents an individual animal, and the bar represents the mean. One-way ANOVA with Sidak correction was used for **(B)**, and Mann-Whitney was used for **(C, D)**.

ABCs are defined differently across studies. CD21-CD23- B cells are a heterogeneous population that includes CD11c+ B cells. We investigated the T-bet expression in B cell subsets since T-bet expression is one of the markers of human circulating ABCs (25). T-bet expression was not observed in conventional B2 B cells (FO and marginal zone) but most CD11c+ CD21-, CD23- B cells express T-bet (**Figure 1A**). There are few T-bet+ cells detected in CD11c- CD21-, CD23- B cells (**Figure 1A**). Therefore, we defined CD11c+, T-bet+, CD21-, and CD23- B cells as murine ABCs and focused on this population for the current study. In human, ABCs have memory-like phenotypes with IgG expression (1). We wanted to know whether ABCs are class-switched in TC mice, and ABCs display isotype heterogeneity; the majority of ABCs are IgD^hi^/IgM+, and there are few IgD-/IgM- cells, which are IgG+. The frequency of class-switched B cells is higher in ABCs than FO B cells (**Figure 1B**). Since the majority of ABCs are IgD^hi^/IgM+ (70%), we decided to compare ABCs to FO B cells.

ABCs are known to express an increased level of MHC II and co-stimulatory molecules compared to FO B cells in aged female mice (2), and we confirmed the higher expression of CD80, CD86 and MHC II in ABCs (**Figure 1C**). We also observed ICOS-L expression, which delivers a positive signal for Tfh differentiation, in ABCs and not in FO B cells. CXCR5 expression on ABCs is a topic of debate (11, 14). We found ABCs express much lower levels of CXCR5 than FO B cells (**Figure 1C**).

In addition to differences in expression of co-stimulatory molecules, cytokine production by ABCs was distinct that observed in FO B cells. We measured cytokines in the supernatant from isolated ABCs or FO B cells. There are reports that secretion of TNFα, IL-4 and IL-10 from ABCs in aged mice (11, 26). We observed increased IL-10 but not TNFα or IL-4 in ABCs compared to FO B cells from TC mice. We also did not find an increased production of Th1 type inflammatory cytokines, IFNγ or IL-12, in the supernatants of ABCs although both are positively regulated by T-bet in T cells. Instead, IL-1β and IL-6 were highly produced by ABCs compared to FO B cells (**Figure 1D**). These data suggest that ABCs are activated B cells, have a distinct cytokine profile, and may induce T cell activation/differentiation more efficiently than FO B cells in TC mice.

### Distinct transcription profile in pathways related to antigen presentation, phagocytosis and metabolism in ABCs

**Figure 2:**
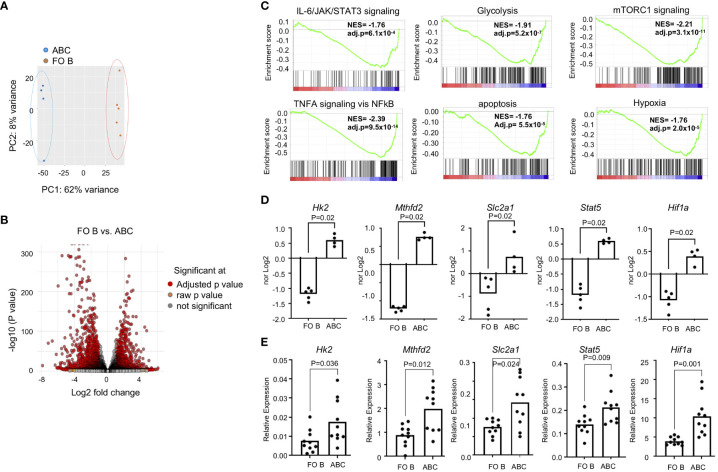
ABCs have a distinct transcription identity. ABCs and FO B cells were isolated, and transcriptome was assessed by RNA-Seq. **(A)** PCA was performed and first and second principal components are plotted. **(B)** Volcano plot was made from the data. **(C)** Gene set enrichment analysis was made from the gene expression data set from B and enrichment score was plotted. **(D)** Normalized expression was plotted from the gene expression data from **(B, E)** Gene expression was performed by qRT-PCR in a separate cohort. Relative expression of each gene of interest was calculated as a relative expression of reference gene, *Polr2a*. (n=3). Each dot represents an individual mouse, and the bar represents the mean. Unpaired Mann-Whitney was used for statistical test.

To understand further the functional difference between FO B and ABCs, we compared their transcriptomic profiles. FO B cells and ABCs were purified and bulk RNA-Seq analysis was performed. Principal component analysis (PCA) analysis revealed a clear separation between FO B cells and ABCs (**Figure 2A**). The volcano plot showed that 1716 genes are up-regulated, and 1595 genes are down-regulated in ABCs compared to FO B cells (log2-fold change >1 or <-1, adj p-value <0.05) (**Figure 2B**). Pathway analysis was performed by fGSEA using MsigDB’s hallmark set. Genes involved in membrane invagination (**Supplementary Figure 1**) and phagocytosis (data not shown) were differentially expressed in ABCs compared to FO B cells, as were genes involved in apoptosis, cytokine signaling (TNFα, and IL-2/STAT5) and metabolism (mTORC1, glycolysis, and hypoxia) (**Figures 2C, D**). Selected RNA-Seq data were confirmed by qRT-PCR in a separate cohort (**Figure 2E**). Gene expression data suggest that ABCs are more effective antigen presenting cells with phagocytic activity. They also have a distinct metabolic program, characterized by increased glycolysis and a hypoxia response, and an increased proinflammatory cytokine/cytokine signaling compared to FO B cells.

### Increased antigen uptake by ABCs

**Figure 3:**
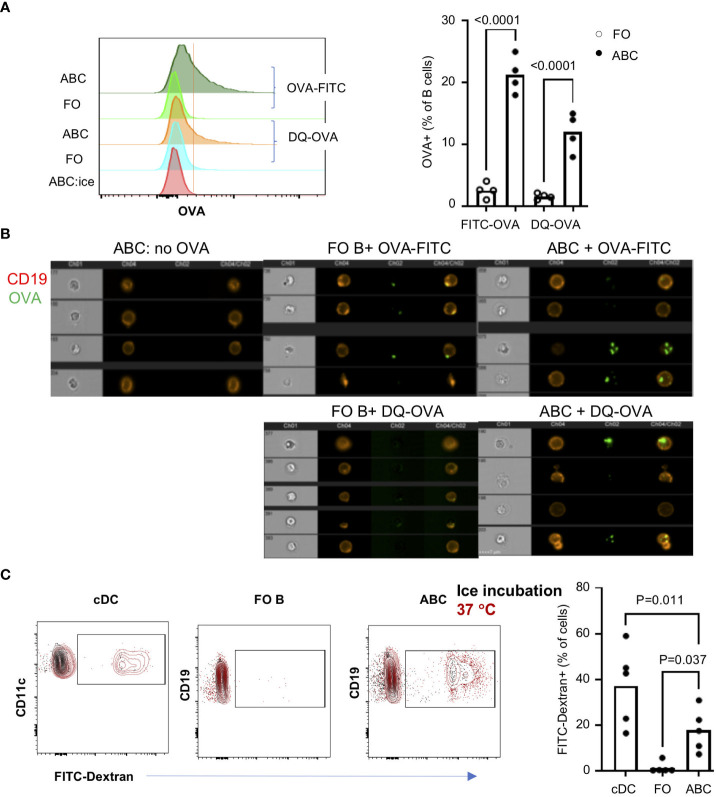
ABCs not FO B cells uptake soluble antigens. ABCs and FO B cells were isolated and incubated alone or with 10 ug/ml of OVA-FITC or DQ-OVA for 30 min at 37°C. To exclude the non-specific binding of OVA, ABCs were incubated with OVA-FITC on ice for 30 min (ABC:ice). After the incubation, OVA signal was detected and analyzed by flow cytometry or image stream. **(A)** A representative overlayed histogram image from the flow cytometry analysis is on the left side and the percent of OVA-positive cells is plotted on the right side. Each dot represents an individual mouse (value was an average of technical duplicates) (n=4). **(B)** OVA location was analyzed by image stream. 4 representative images from each condition are presented. **(C)** FITC-Dextran (10 μg/ml) were incubated with splenocytes for 30 min at 37°C or on ice. After incubation, cells were stained with cell lineage surface markers, and the percent of FITC-Dextran positive cells was analyzed by flow cytometry. Each dot represents individual mouse (value was an average of technical duplicates) and the bar represents the mean (n=2). One-way ANOVA was used for statistics and Bonferroni correction was applied for multiple comparison.

The frequency of B cells recognizing a specific antigen is very low, usually less than 0.05% in the normal repertoire (27). Enrichment of genes involved in phagocytosis, autophagy, and membrane invagination led us to investigate whether ABCs have antigen uptake mechanisms other than the BCR-dependent mechanism. Innate immune cells uptake soluble antigens through antigen independent mechanisms including phagocytosis, Fc-dependent endocytosis, and pinocytosis [reviewed in (28, 29)], but B cells, in general, internalize antigens through a direct interaction between the antigen and the BCR. We asked whether a high percent of ABC would uptake a particular antigen, ovalbumin (OVA). Each B cell subset was incubated with FITC-OVA [~ 3 nm hydrodynamic size (30)], and OVA+ B cells were enumerated. B cells were also incubated with DQ-OVA which enabled us to identify OVA in the lysosome to assess peptide processing of the ingested OVA protein in B cells. Approximately 25% and 15% of ABCs were positive for FITA-OVA or DQ-OVA, respectively compared to less than 5% of FO B cells (**Figure 3A**). We confirmed the intracellular location of OVA by image stream (**Figure 3B**). Then, we assessed uptake of another antigen, FITC-Dextran (1 nm diameter) by ABCs. Classical dendritic cells (cDCs: lin-CD11c^hi^B220-MHCII^hi^) were included as a positive control for phagocytic activity, approximately 40% of cDCs ingested FITC-Dextran. We detected FITC-Dextran in approximately 20% of ABCs and less than 5% of FO B cells (**Figure 3C**). Uptake of FITC-Dextran or FITC-OVA did not occur when cells were incubated on ice confirming that positivity did not represent membrane binding. Both OVA and Dextran are small molecules that can be ingested by pinocytosis. To address whether OVA uptake and T cell activation is ABC lineage-specific or results from inflammatory conditions in lupus mice, we investigated OVA uptake by ABCs from aged female C57BL/6J mice. A significant amount of OVA was ingested by ABCs from non-lupus mice (**Supplementary Figure 2A**). However, we did not detect T cell activation or Tfh differentiation by OVA+ABCs from non-autoimmune mice (**Supplementary Figure 2B**).

The differentially expressed genes seen by RNA-Seq suggest several phagocytic receptors may be expressed in ABCs. We confirmed the expression of CD36 by qRT-PCR and surface expression of CD36 by flow cytometry (**Supplementary Figure 3A, B**). Expression of CD36 suggests that ABCs might internalize antigens by phagocytosis. We, therefore, asked whether CD36 expression is required for OVA uptake by ABCs. Co-staining with CD36 and OVA-FITC showed that OVA was taken up in both CD36+ and CD36- ABCs (**Supplementary Figure 3C**). Moreover, blocking CD36 by neutralizing antibodies did not affect OVA uptake (**Supplementary Figure 3D**). We also tested whether ABCs were able to phagocytose a large particle (typically 1-2 µm), Zymosan. Less than 5% of ABCs exhibited phagocytic activity (data not shown). These data suggest that ABCs can uptake small antigens by a non-phagocytic mechanism, and CD36 does not participate in this process.

**Figure 4:**
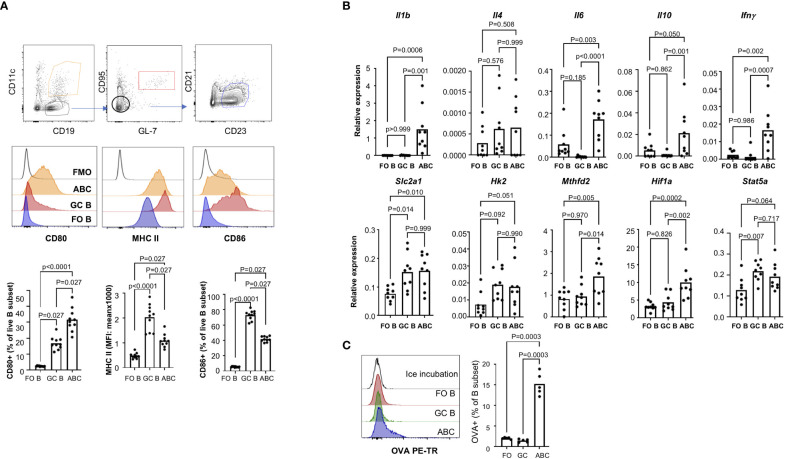
ABCs have distinct cytokine production and antigen uptake. **(A)** Surface expression of MHC II, CD86 and CD80 was compared among the FO B, GC B and ABCs. Representative images of each population and overlayed histograms of each molecule are presented and summary graphs are on the bottom row (n=3). **(B)** Expression of cytokines and metabolic genes was measured by qRT-PCR, and relative expression was calculated and plotted (n=3). **(C)** OVA uptake was measured by flow cytometry. A representative overlayed histogram and the percentage of OVA+ B cells was plotted (n=3). One-way ANOVA was used for statistics and Sidak’s correction was applied for multiple comparison.

Since ABCs exhibit phenotypes of activated B cells, we compared ABCs with GC B cells which are antigen activated B cells. GC B cells (CD95+GL7+) express high levels of MHC II and CD86 but not CD80 (**Figure 4A**). Cytokine expression from FO B, GC B, and ABCs showed more significant difference between GC B and ABCs as shown in **Figure 4B**. Similar to FO B cells, GC B cells showed little expression of proinflammatory cytokines (*Il1b*, *Il6* and *Ifng*). Both ABCs and GC B cells have an increased expression of genes involved in glycolysis and *Stat5* compared to FO B cells, but *Hif1a* expression was increase only in ABCs. Lastly, we compared OVA uptake and GC B cells that show low OVA uptake compared to ABCs (**Figure 4C**).

### ABCs induce stronger T cell activation and promote Tfh differentiation

**Figure 5:**
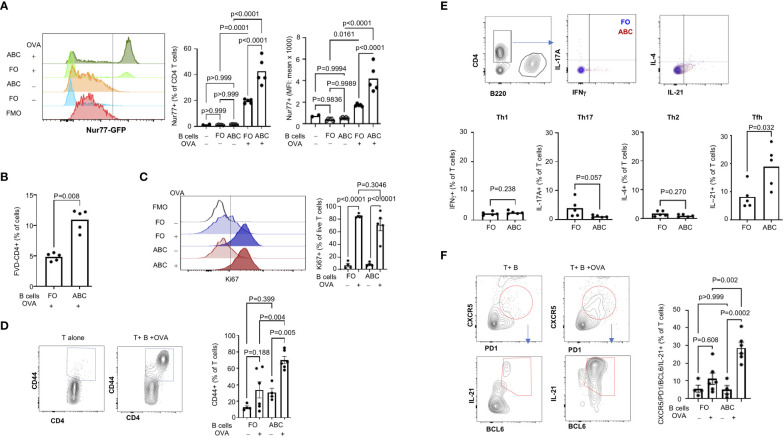
ABCs induce stronger T cell activation and Tfh differentiation. **(A)** TCR activation was measured by GFP expression in naïve CD4+ T cells (from OTII. Nur77-GFP) after 16 hours incubation with B cells ± OVA as indicated in the figure. A representative overlayed histogram of Nur77-GFP is on the left and the percent of Nur77-GFP positive and MFI of Nur77-GFP positive T cells are plotted on the right (n=3). **(B)** T cells were co-cultured with B cells for 4 days, and the live T cells were identified as FVD-negative CD4+ T cells. **(C, D)** T cell proliferation and activation were measured by Ki67 expression and CD44 expression in live CD4+ T cells from 4-day culture with B cells. Ki67 positivity was gated relative to FMO and the CD44 positive signal was calculated by culture of T cells alone (unstimulated condition) (n=3). **(E)** Naïve CD4+ T cells were cultured with FO B or ABCs (± OVA) for 4 days. Cytokine expression was measured by intracellular staining using flow cytometry. The percent of cytokine-positive T cells was plotted. Each dot represents an individual animal (each value was an average of technical duplicates) and the bar represents the mean (n=2). **(F)** Naïve CD4+ T cells were cultured with either FO B or ABCs (± OVA) for 4 days. Tfh cells were identified by expression of CXCR5/PD-1/BCL6/IL-21 by flow cytometry. Representative overlayed flow images are on the left and the percent of CXCR5/PD1+ T cells and the percent of Tfh cells are plotted on the right (n=3). Each dot represents an individual animal, and the bar represents the mean. One-way ANOVA was used for statistics and Sidak’s correction was applied for multiple comparisons.

Next, we wanted to address whether ABCs play a role in T cell activation as APCs. First, we asked if the ABCs can activate T cells through the T cell receptor (TCR). We compared Nur77 induction in T cells, an immediate downstream molecule in TCR signaling. The expression of Nur77 is positively correlated with TCR strength and is not induced by non-TCR signaling such as signaling through cytokine receptors (17, 31). Naïve CD4+ T cells from OTII.Nur77-GFP mice were cultured with ABCs or FO B cells, pre-incubated with or without OVA protein. ABCs induced a stronger TCR signal, assessed by expression of Nur77-GFP in naive CD4 T cells (**Figure 5A**) and T cell activation was OVA dependent, as there was minimal Nur77 induction by ABCs in the absence of OVA.

Next, we assessed T cell proliferation/activation induced by ABCs or FO B cells. After 3 days of co-culture, there were more live T cells in the culture containing ABCs with OVA than FO B cells with OVA (**Figure 5B**). Both FO B cells and ABCs induced Ki67 expression in live CD4+ OTII T cells in the presence of OVA protein (**Figure 5C**). Although there was no difference in the frequency of Ki67+ T cells, an increased number of CD44+ T cells was observed by co-culture with ABCs (**Figure 5D**). Next, we compared effector T (Teff) differentiation by OVA-presenting ABCs or FO B cells. ABCs induced little Th1, Th2 or Th17 differentiation and no difference was observed between ABCs and FO B cells (**Figure 5E**). In contrast, ABCs induced more IL-21-positive T cells than FO B cells (**Figure 5E**). IL-21 is a key cytokine of Tfh (32). PD-1, CXCR5 (a surface marker of Tfh) and BCL6 (a master transcription factor of Tfh) expression were measured in T cells activated by ABCs or FO B cells to confirm that IL-21 producing T cells were Tfh; CXCR5+/PD-1+/BCL6+/IL-21+ cells were induced by ABC/OVA-primed T cells but not by FO B OVA-primed T cells (**Figure 5F**). These data suggest that ABCs can promote preferential differentiation of naïve T cells to Tfh and cognate antigen presentation by ABCs is required.

### ABCs exhibit increased glycolysis, mitochondrial respiration and ROS production

**Figure 6:**
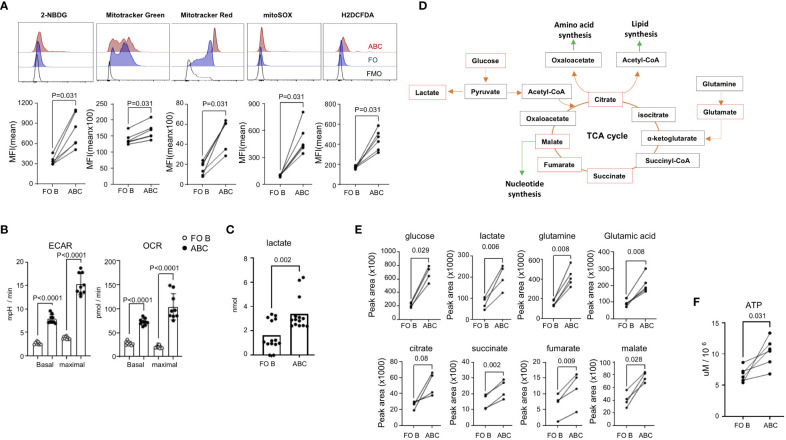
Increased energy metabolism and ROS production in ABCs. **(A)** Spleen cells from TC mice were stained with 2-NBDG, Mitotracker Green, Mitotracker Red, MitoSOX, or H2DCFDA. Cells were washed, and antibodies were incubated to identify each B cell subset. MFI was calculated from each B cell population and plotted. Each dot represents an individual animal (the value was an average of technical duplicates) (n=3). **(B)** 2x10^5^ isolated FO B or ABCs were plated in the seahorse chamber and ECAR and OCR were measured. Basal respiration and maximal respiration were obtained and plotted. Each dot represents an individual animal (the value was an average of technical triplicates) and the bar represents the mean (n=3). **(C)** Isolated FO B or ABCs were cultured overnight (10^6^/ml and 100 ul/well), and lactate level in the supernatant was measured. Each dot represents an individual animal, and the bar represents the mean (n=6). **(D)** Diagram of TCA cycle and linked anabolic pathway. An untargeted metabolomic profile was measured from isolated B cells, and the metabolites which were increased in ABCs over FO B cells are labeled by a red box. **(E)** Peak area value of each metabolite in FO B or ABCs is plotted. Each dot represents an individual animal. **(F)** FO B cells and ABCs were isolated and intracellular ATP level was measured. Each dot represents an individual animal (n=3). One-way ANOVA was used for statistics and Sidak’s correction was applied for multiple comparisons for **(B)**. Unpaired Mann-Whitney was used for **(C)**. Paired Wilcoxon test was performed for **(A, E, F)**.

In addition to the enrichment of antigen presentation and phagocytic markers, ABCs display a significant difference in metabolism (in **Figure 2**). To confirm increased energy metabolism in ABCs, we measured glucose uptake and mitochondrial metabolism. 2-NBDG (2-[*N*-(7-nitrobenz-2-oxa-1,3-diazol-4-yl) amino]-2-deoxy-D-glucose) is a fluorescent analogue of D-glucose which enables the measurement of glucose uptake in a quantitative manner (33). Consistent with the increased level of *Slc2a1* (the gene encoding Glut1) seen in the RNA-Seq analysis, we found that significantly more 2-NBDG was taken up by ABCs than FO B cells (**Figure 6A**). Mitochondrial mass and mitochondrial membrane potential were measured by MitoTracker Green and MitoTracker Red, respectively. ABCs exhibited slightly increased mitochondrial mass compared to FO B cells and displayed enhanced mitochondrial membrane potential. Increased mitochondrial mass was confirmed by measuring the mitochondrial genes, *Nd1* and *Rnr2* (**Supplementary Figure 4A**). Moreover, ABCs exhibited higher levels of reactive oxygen species (ROS) measured by MitoSOX and H2DCFDA (**Figure 6A**), causing an increased lipid peroxidation in ABCs (**Supplementary Figure 4B**). Stimulation of B cells either by antigen (anti-IgM) or a TLR7 agonist (R848) increased glucose uptake and cellular ROS production in both B cell subsets (**Supplementary Figure 5A**). We repeated the experiment in B6.lpr mice, another lupus-prone strain, and observed increased metabolic activity in ABCs compared to FO B cells (**Supplementary Figure 5B**). An increased mitochondrial ROS was observed in ABCs, but glucose uptake and mitochondrial membrane potential were not different between unstimulated ABCs and unstimulated FO B cells in aged female non-autoimmune mice (**Supplementary Figure 5C**). These data suggest a metabolic difference (increased glycolysis, mitochondrial membrane potential and ROS production) in ABCs could be driven by the autoimmune background.

We confirmed the data obtained through flow cytometry analysis by seahorse technology, which allows for simultaneous kinetic measurement of cellular respiration and glycolysis. ABCs from TC mice showed increased oxygen consumption rate (OCR) and extracellular acidification rate (ECAR) at basal state and at the point of maximal respiration compared to FO B cells (**Figure 6B**). Increased glycolysis in ABCs was confirmed by increased lactate production, which is a final product of glycolysis (**Figure 6C**).

We further characterized the metabolites in the ABCs and FO B cells using LC-MS/MS analysis. An equal number of ABCs and FO B cells were isolated, and an untargeted metabolome analysis was performed. PCA analysis was carried out using an unsupervised dimensionality reduction method. There was a clear spatial separation between the two groups (**Supplementary Figure 6**). Fold change and t-test were combined to screen for significantly different metabolites between the groups. Those with a p-value of less than 0.05 and log2(FC) >1 were considered statistically different. The results showed that 87 of 145 metabolites were elevated in ABCs. The heat map of the top 50 differential metabolites is presented in **Supplementary Figure 6**. Many of these metabolites function within the glycolysis and TCA cycle pathways (**Figures 6D, E**). Increased metabolites may result from high energy production or from blocking of a downstream pathway, leading to cell death. To confirm that the increased metabolic status was indeed related to increased energy production in ABCs, we measured the level of intracellular ATP, which was higher in ABCs than FO B cells (**Figure 6F**). These data suggest that ABCs are metabolically hyperactivated compared to FO B cells.

### Metformin suppresses antigen uptake and Tfh differentiation by ABCs

**Figure 7:**
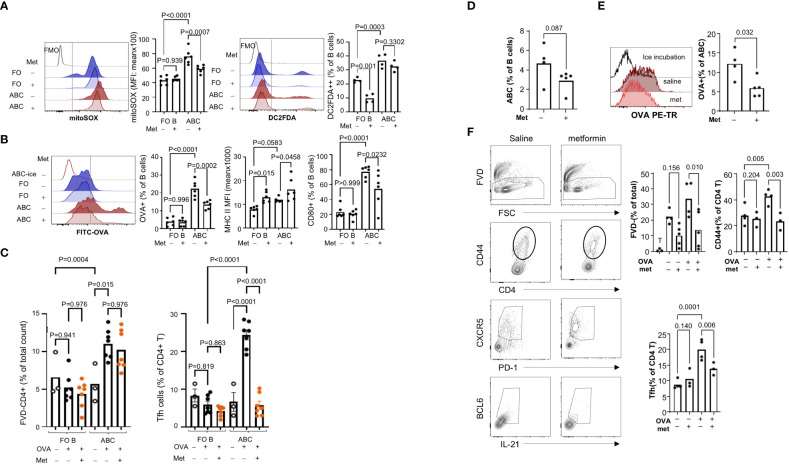
Metformin treatment suppressed OVA uptake by ABCs and subsequent Tfh differentiation. **(A)** ABCs and FO B cells were cultured alone or with metformin (1 mM) overnight. After the culture, mitochondrial ROS production and total ROS production were measured by MitoSOX and H2DCFDA staining. MFI was analyzed by flow cytometry and the value was plotted. Each dot represents an individual animal (value was an average of technical duplicates) and the bar represents the mean (n=4). **(B)** B cells were isolated and cultured alone or with metformin overnight. After the incubation, cells were washed, and incubated with FITC-OVA for 30 min at 37°C or on ice. Surface staining of MHC II and CD80 was performed after FITC-OVA incubation. Each dot represents an individual animal, and the bar represents the mean (n=4). **(C)** FO B cells or ABCs were pre-treated with metformin for 3 hours prior to incubation with OVA. After OVA incubation, naïve CD4+ T cells, which were isolated from OTII mice, were added to B cells and cultured for 4 days. The percentage of live T cells and Tfh cells (CXCR5+/PD1+/BCL6+/IL-21+) was identified by flow cytometry and plotted. Each dot represents an individual animal, and the bar represents the mean (n=3). One-way ANOVA was used with Sidak’s correction for multiple comparisons. TC mice were treated with metformin or saline for one week and spleens were collected for ABCs isolation. **(D)** Percent of ABCs was plotted (n=4). **(E)** ABCs were isolated and OVA uptake was measured by flow cytometry. OVA+ ABCs was calculated based on the ice incubation control (n=4). **(F)** ABCs were isolated and OVA protein was incubated for 3 hours. Then, naïve CD4+ T cells from OT II mice and ABCs were co-culture for 4 days. Viability, CD44+ T cells and Tfh cells were measured as described in **(C)** (n=4). Each dot represents an individual animal, and the bar represents the mean. One-way ANOVA was used for statistics and Sidak’s correction was applied for multiple comparisons.

Hypermetabolism is closely linked with altered function and differentiation of leukocytes [reviewed in (34)]. Several reports showed that treatment of lupus-prone mice with metabolic modulators can prevent disease development or even reverse the established disease phenotypes (35). Since ABCs display increased energy metabolism, we wanted to address whether targeting mitochondrial metabolism and ROS production might decrease ABC-mediated Tfh differentiation. Metformin is a drug with pleotropic effects, including inhibition of mitochondrial respiratory chain complex I and production of ROS (36). In B cells, metformin can block terminal differentiation to plasma cells (37), but the effect on ABCs is not known. Because the receptor for metformin in B cells is not known, we measured the expression of metformin receptors in B cells. ABCs express *Slc22a3* but no *Slc22a1* or *Slc22a2* and the level of *Slc22a3* was higher in ABCs compared to FO B cells (**Supplementary Figure 7A**). First, we confirmed the effect of metformin on mitochondrial ROS production in B cells; metformin decreased mitochondrial ROS, but not total ROS, in ABCs (**Figure 7A**). Next, we determined whether metformin could modulate internalization of FITC-OVA and co-stimulatory molecule expression in ABCs. OVA-positive B cells with or without metformin treatment were quantified by flow cytometry; metformin treated ABCs showed a significant decrease in OVA uptake (**Figure 7B**). The inhibition of OVA uptake was also observed when ABCs were treated with phenformin which is a metformin-related drug (**Supplementary Figure 7B**), that is transferred into cells in a non-receptor dependent fashion (38). Metformin treatment did not decrease the surface expression level of MHC II but did decrease CD80 expression in ABCs (**Figure 7B**). Suppression of OVA uptake and CD80 expression by metformin led us to investigate whether metformin altered T cell activation. It did not lead to decreased T cell viability, but no longer induced Tfh differentiation (**Figure 7C**). We next investigated the effect of metformin on ABC function *in vivo*. A study by Lee and colleagues showed a significant reduction of most B cell subsets in Roquin*^san/san^* lupus mice by 3 weeks metformin treatment (37). To avoid the loss of ABCs by long-term treatment with metformin, we treated TC mice with metformin for 1 week, and assessed the function of ABCs. We did not find a significant difference in body weight, number of splenocytes or percentage of B cell subsets in metformin treated TC mice compared to saline treated TC mice (data not shown). We observed a trend of decrease in percentage of ABCs and further decreased OVA uptake observed by ABCs from metformin treated TC mice (**Figures 7D, E**). The ABCs from metformin-treated TC mice also showed a decreased ability of T cell activation and Tfh differentiation. Overall, these data suggest that metformin treatment inhibits antigen uptake and suppresses Tfh differentiation by ABCs.

## Discussion

Our study shows that ABCs from lupus prone TC mice exhibit a distinct gene expression profile which is specifically enriched in pathways of antigen-uptake, cytokine signaling, and energy metabolism. ABCs engage in active uptake of soluble antigens and express high levels of co-stimulatory molecules for T cell activation, thereby inducing T cell activation skewed toward Tfh differentiation. Moreover, *in vivo* and *in vitro* treatment with metformin decreased antigen uptake by ABCs and subsequent Tfh differentiation.

B cells can contribute to the development of autoimmune disease in various ways. The most prominent way is by the production of self-reactive antibodies which target the host’s organs (39). Indeed, several studies have shown that ABCs are able to secrete antibodies after activation. ABCs from lupus-prone mice or virus infected animals produce self-reactive antibodies or anti-viral antibodies, respectively (10, 12). Depletion of ABCs leads to a reduction of autoantibodies in the serum of lupus prone mice (3). Compared to FO B cells, which express low levels of costimulatory molecules, ABCs express costimulatory molecules and higher levels of MHC II. This phenotypic characterization suggests that ABCs can activate T cells more efficiently than naïve B cells. Marrack and colleagues showed that CD11c+ B cells express distinct chemokine receptors, reside at the T-B border and activate T cells in aged female mice as well as in *B6.Nba2* lupus mice (14). Li and colleagues found that CD11c+ B cells are required for the expansion of Tfh cells in B cell specific SHIP-deficient lupus mice (15). Our study confirmed ABCs act as efficient APCs leading to an activating antigen-specific T cell. OVA uptake maybe a common function of ABCs, since ABCs from aged non-lupus mice also performed this function. Interestingly, the antigen-specific T cell activation was much weaker in non-lupus mice. It may reflect a difference of OVA processing or cytokine production between lupus mice and non-lupus mice.

Our study confirmed several features of ABCs, including the increased expression of costimulatory molecules and antigen uptake needed for T cell activation and an increased frequency of class-switched ABCs in lupus-prone TC mice. Although there was an increase in class-switched populations (~10% of total ABCs), most ABCs express high levels of IgD and IgM. It is important to understand whether those class-switched ABCs and IgD^hi^IgM+ ABCs have different functions and whether any of subset contributes to lupus development or progression. A recent study characterized CD11c+ ABCs in the MRL/lpr mouse model of lupus (40). In this study, heterogeneity of ABCs was found with respect to expression of integrins and memory or plasmablast markers. Many genes expressed in ABCs in this study were also increased in ABCs in our study. Our data also revealed additional pathways that are enriched in ABCs, including amino acid transporters and cytokine receptor signaling.

We also made some novel observations, such as an increased hypoxic pathway, increased mitochondrial metabolism and mitochondrial ROS production, along with preferential induction of Tfh cells. T cell activation and Tfh differentiation by ABCs may reflec in part an autoimmune background since this function was markedly diminished in ABCs from non-lupus background. Since OVA uptake occurs similarly in ABCs from both lupus and non-lupus mice, antigen processing may differ depending on the genetic background of strain. It needs to be determined whether OVA is taken into an endo-lysosomal compartment in ABCs in lupus mice but not in non-lupus mice. Comparison of ABCs to GC B cells highlighted the uniqueness of ABCs as APCs. GC B cells express higher levels of MHC II and CD86, but CD80 expression is much lower in GC B cells than ABCs. The cytokine profile of ABCs and GC B cells are different; GC B cells do not produce IL-10 or IL-1β. Both GC B cells and ABCs express high levels of genes involved in glycolysis, suggesting both B cell types have enhanced glycolysis. An increased levels of Hif1a and Mthfd2 expression was observed only in ABCs, suggesting a metabolic difference between these two activated B cell populations.

ABCs have elevated expression of genes involved in phagocytosis, antigen presentation and plasma membrane invagination. CD36 and AXL are cell surface molecules on ABCs, involved in antigen uptake in innate immune cells and have been suggested to help clear apoptotic debris in DEF6/Swap70 double knockout mice (41). CD36 is a scavenger receptor and facilitates uptake of fatty acids (42). This is a key step in energy metabolism and may explain the increased lipid metabolites in ABCs (**Supplementary Figure 4**). In line with a role of CD36 in lipid metabolism, a recent study showed an expansion of T-bet+ B cells in adipose tissue where they contribute to the metabolic disorder in obese mice (43). Despite their high level of CD36 expression, CD36 was not responsible for uptake of OVA or dextran.

Another observation we made was the metabolic difference between ABCs and FO B cells. Human circulating ABCs are suggested to be atypical memory B cells, and memory B cells are relatively quiescent metabolically. AMPK appears to be important in mitochondrial homeostasis and maintaining memory B cells (44). Therefore, we hypothesized that CD11c+ ABCs might utilize mitochondria or lipid oxidation for their energy production. In contrast to this hypothesis, ABCs exhibit increased metabolism in both mitochondrial respiration and glycolysis compared to naive FO B cells even without ex vivo stimulation. This is consistent with a recent study of the bm2 mouse model of SLE. The investigators showed an increased glycolytic capacity of ABCs in this mouse: moreover, inhibition of heightened glycolysis by 2-DG administration inhibited ABC formation and decreased the autoantibody production (45), suggesting that the glycolytic pathway is required for ABC generation, ABC survival or antibody production by ABCs in lupus mice. This phenotype was observed in ABCs from another lupus mouse model, B6.lpr, but not in ABCs from aged female mice from a non-lupus background. We also compared metabolic status in ABCs and FO B cells from NZB/W F1 lupus mice; enhanced glucose uptake and mitochondrial activation were observed in ABCs compared to FO B cells (data not shown). Increased mitochondrial ROS production was observed in ABCs from both autoimmune and non-autoimmune mice. These data suggest that a disease-dependent metabolic adaptation in ABCs exists. It is unclear whether the metabolic difference in ABCs between lupus mice and aged non-lupus mice is due to a different cytokine milieu in diseased and in healthy aging or different precursors (naïve B cells or memory B cells) or different genetic influences. Intermediate levels of ROS can serve as signaling molecules and are important for lymphocyte activation augmenting intracellular signaling pathways downstream of BCR (46). Elevated ROS can modify intracellular molecules and oxidized mitochondrial DNA which can be translocated into cytoplasm, leading to an activation of the inflammasome and IL-1β secretion in macrophages (47). We observed an increased secretion of IL-1β by ABCs, which may be due to an elevation of ROS and increased inflammasome activity.

The observation that metformin treatment prevents antigen uptake and Tfh differentiation is novel, too. *In vitro* treatment with metformin (few hours) did not affect the expression of co-stimulatory molecules or cytokine production. *In vivo* exposure to metformin (1 week) did not change the frequency of B cell subsets, including FO B, MZ B, GC B and CD138^++^ PCs in TC mice. We found, however, a trend toward fewer ABCs in metformin treated TC mice. Moreover, those ABCs exhibit reduced OVA uptake and T cell activation capacity. The mechanisms by which metformin alters ABC function need to be further studied. Metformin is a therapeutic option for several diseases. The Met LUPUS trial, an open-labelled clinical trial, showed that metformin reduces disease flares in SLE patients (48). Metformin treatment can suppress disease progression in *Roquin* ^san/san^ mice (37). In TC mice, metformin can prevent disease onset and reverse the disease progression when used with 2-DG, an irreversible inhibitor of glucose transporter (35). The combination of metformin and 2-DG actively suppresses CD4+ effector T cells or memory T cell differentiation/accumulation, but the direct effect of these drugs on B cells was not addressed. Indeed, the mechanism of metformin on B cells or B cell subsets is still an open question. Our study shows that metformin can directly affect the function of ABCs *in vitro* by reducing antigen uptake and CD80 expression, leading to reduced Tfh differentiation. Surprisingly, there was no change in cytokine production or in the level of expression of other costimulatory molecules or MHC II following metformin treatment. The mechanism by which metformin causes the decrease in antigen uptake and CD80 expression requires further study.

## Data availability statement

The datasets presented in this study can be found in online repositories. The names of the repository/repositories and accession number(s) can be found below: https://www.ncbi.nlm.nih.gov/geo/, GSE214185.

## Ethics statement

The animal study was approved by The Feinstein Institutes for Medical Research (FIMR) institutional animal care and use committee (IACUC). The study was conducted in accordance with the local legislation and institutional requirements.

## Author contributions

SK conceived and designed the overall study and interpretated the results. IR performed the experiments and analyzed the data. AS and KL performed the RNA-sequencing analyses. VK designed and performed the metabolomic analyses. SK, IR, YA and BD participated in the interpretation of the study, writing and critical review of the manuscript. All authors contributed to the article and approved the submitted version.
