# Supplementary material for: Phenotypic and functional characteristics of murine CD11c+ B cells which is suppressed by metformin
Source: Front Immunol. 2023 Sep 6;14:1241531. doi: 10.3389/fimmu.2023.1241531 (PMC10512061; doi:10.3389/fimmu.2023.1241531)
Supplement: Supplementary file 1 [file DataSheet_1.pdf]

Supplemental material

Supplementary figure 1: Heat map of relative gene expression from ABCs and FO B cells.

Supplementary figure 2: OVA uptake and T cell activation by ABCs from non-lupus mice.

Supplementary figure 3: CD36 expression is not required for OVA uptake by ABCs.

Supplementary figure 4: Quantification of mitochondrial DNA copy number and oxidized lipid in B cells.

Supplementary figure 5: Metabolic assessment of B cells by flow cytometry.

Supplementary figure 6: Metabolomic analysis of FO B cells and ABCs.

Supplementary figure 7: Metformin receptor expression in B cells and suppression of OVA uptake by metformin and phenformin.

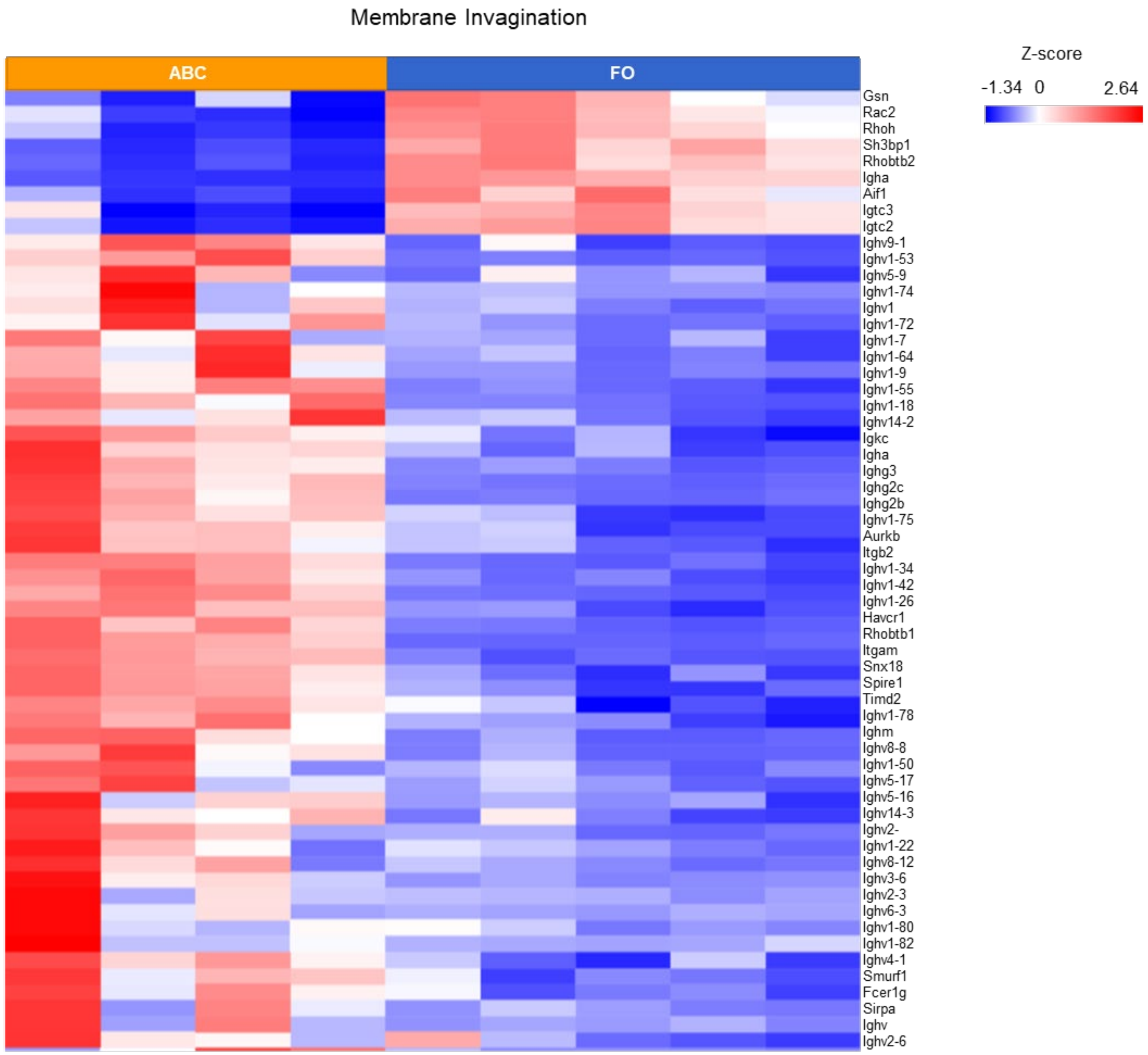

Supplementary figure 1: Heat map of relative gene expression from ABCs and FO B cells.

Het map of Membrane Invagination pathway from RNA-seq analysis. Coloring is based on row Z-score. Each data point (column) represents one mouse.

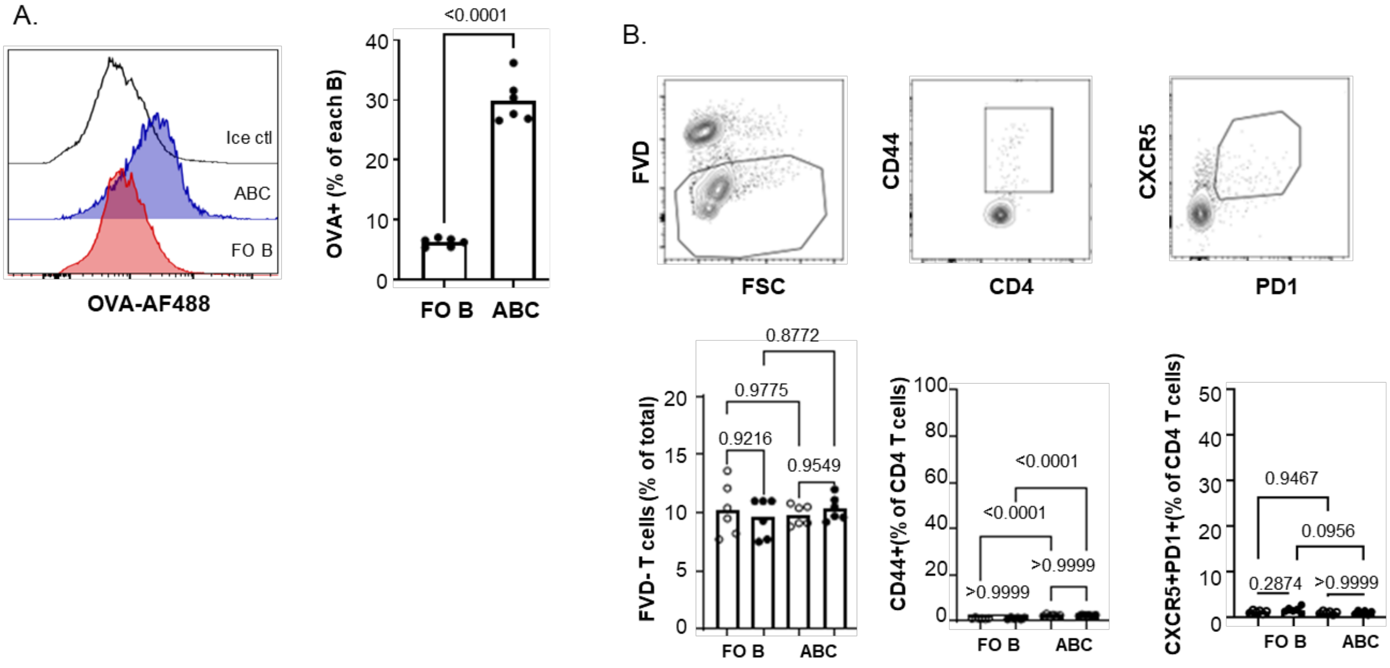

Supplementary figure 2: OVA uptake and T cell activation by ABCs from non-lupus mice. ABCs and FO B cells were isolated from old female (12-14 months old) C57BL/6 mice and functional investigation was conducted. **(A)** OVA uptake by FO B and ABCs was measured by flow cytometry. Overlayed histogram is on the left and the percent of OVA+ B cells was plotted on the right. **(B)** FO B and ABCs were cultured with OT II naïve CD4+ T cells ± OVA protein for 4 days. After the culture, live T cells, CD44+ activated T cells and Tfh-like cells (CXCR5+PD1+) were measured by flow cytometry and plotted. Each dot represents an individual mouse, and the bar represents the mean (n=3). Unpaired Mann-Whitney was used for (A), and one-way ANOVA was used for (B). Sidak's correction was applied for multiple comparisons.

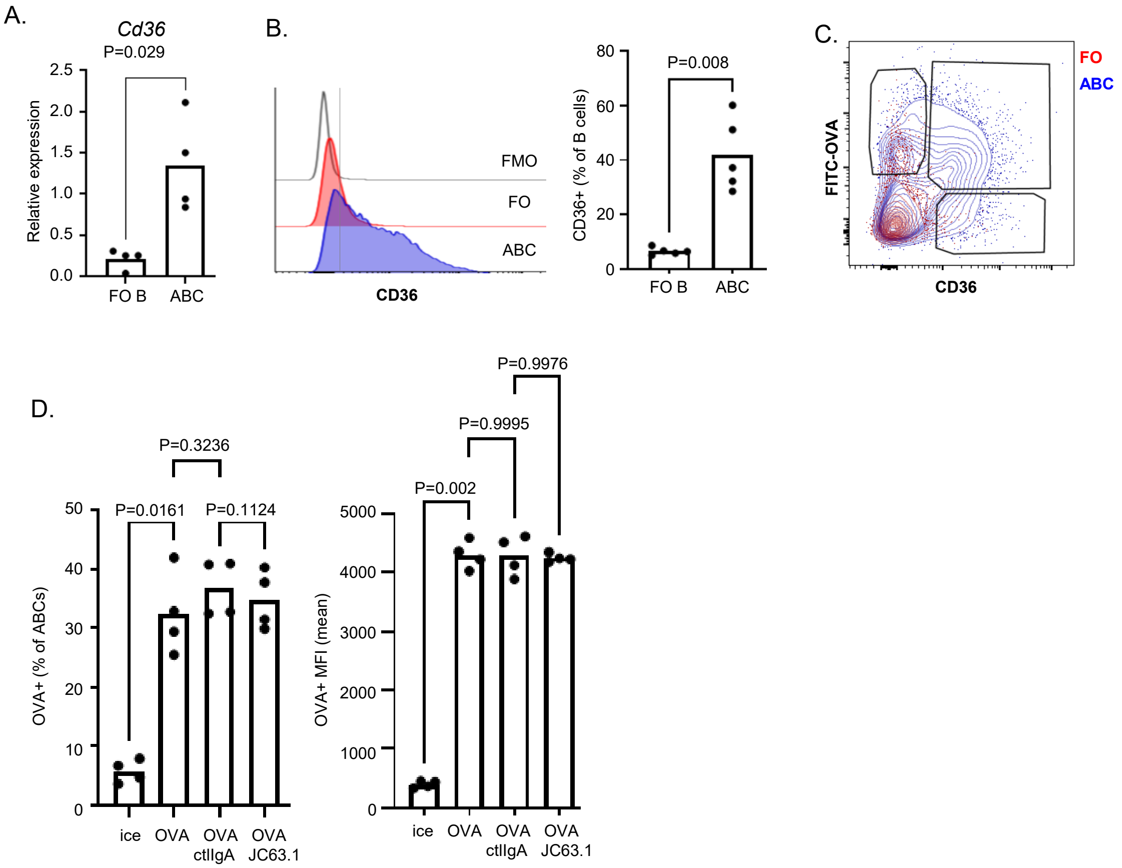

Supplementary figure 3: CD36 expression is not required for OVA uptake by ABCs. Expression of CD36 was measured from the isolated ABCs and FO B cells by qRT-PCR **(A)** and flow cytometry **(B)**. The histogram of CD36 and the percent of CD36 positive cells is plotted in the graph. Each dot represents an individual animal, and the bar represents the mean (n=2). Unpaired Mann-Whitney was used. **(C)** ABCs were isolated and incubated with FITC-OVA (10 μg/ml) for 30 min at 37 °C incubator for uptake. After the incubation, cells were collected and washed with ice-cold staining buffer twice. Then, cells were stained with anti-CD36 antibodies. OVA-positive and CD36-positive cells were analyzed by flow cytometry. A representative contour plot image is presented from 3 independent experiments. (D) Isolated ABCs were incubated with control Ig or anti-CD36 antibodies (JC63.1) for 2 hours. The cells were incubated with OVA protein for 30 min at 37 °C or on ice. The percentage of ABCs and fluorescence intensity of OVA was assessed by flow cytometry and plotted in the graphs. Each dot represents an individual animal (n=2). Unpaired Mann-Whitney was used for (A-B), and one-way ANOVA was used for (D). Sidak's correction was applied for multiple comparisons.

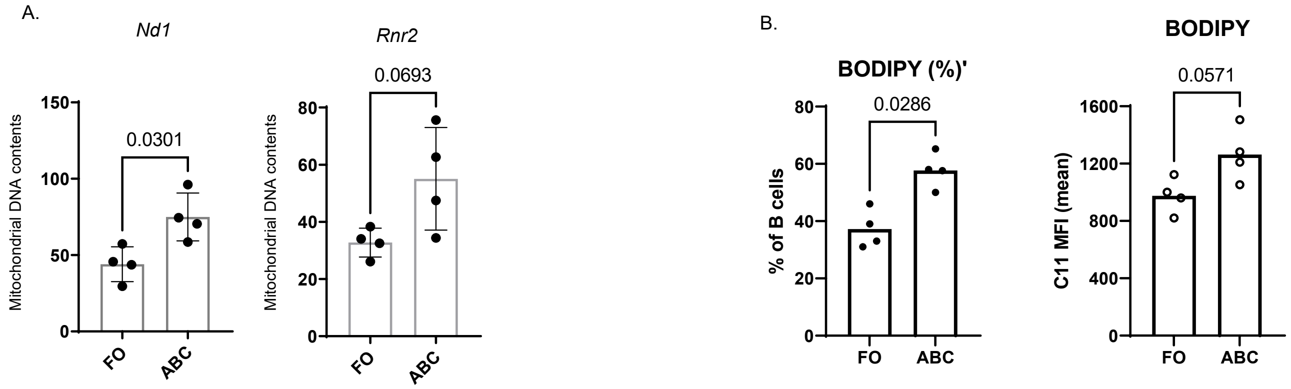

Supplementary figure 4: Quantification of mitochondrial DNA copy number and oxidized lipid in B cells. (A) Mitochondrial DNA was quantified from isolated ABCs or FO B cells by measuring mt-DNA (*Nd1* and *Rnr2*) and nu-DNA (*Actin*) by qPCR. The relative amount of mitochondrial DNA contents was calculated as described in the methods. **(B)** Isolated ABCs and FO B cells were incubated with C11-BODIPY, and the oxidized lipid was measured by quantifying fluorescence by flow cytometry. Each dot represents an individual mouse (n=4). The bar graph represents the mean. Unpaired Mann-Whitney was used.

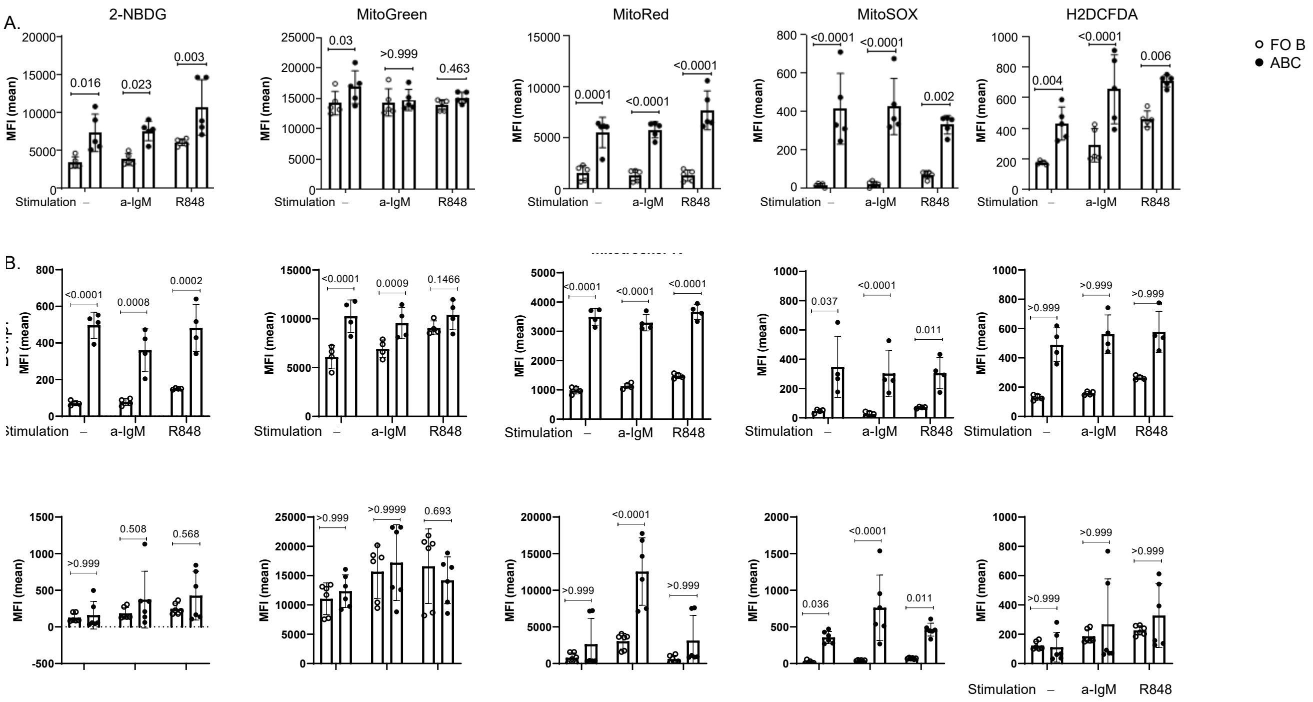

Supplementary figure 5: Metabolic assessment of B cells by flow cytometry. Spleens were harvested TC (A), B6.lpr (B), or C57BL/6 (C) mice. FO B cells and ABCs were isolated and cultured with or without B cell stimulators (anti-IgM: 10 μg/ml or R848: 100 ng/ml) as indicated in the figure for 18 hours. After the culture, cells were stained with 2-DBDG, MitoTracker Green, MitoTracker Red, MitoSOX, or H2DCFDA as described. Cells were washed to remove unincorporated dye and dead cells were excluded by FVD staining. MFI was calculated from each B cell population and plotted. Each dot represents an individual animal (the value was an average of technical duplicates) (n=3).

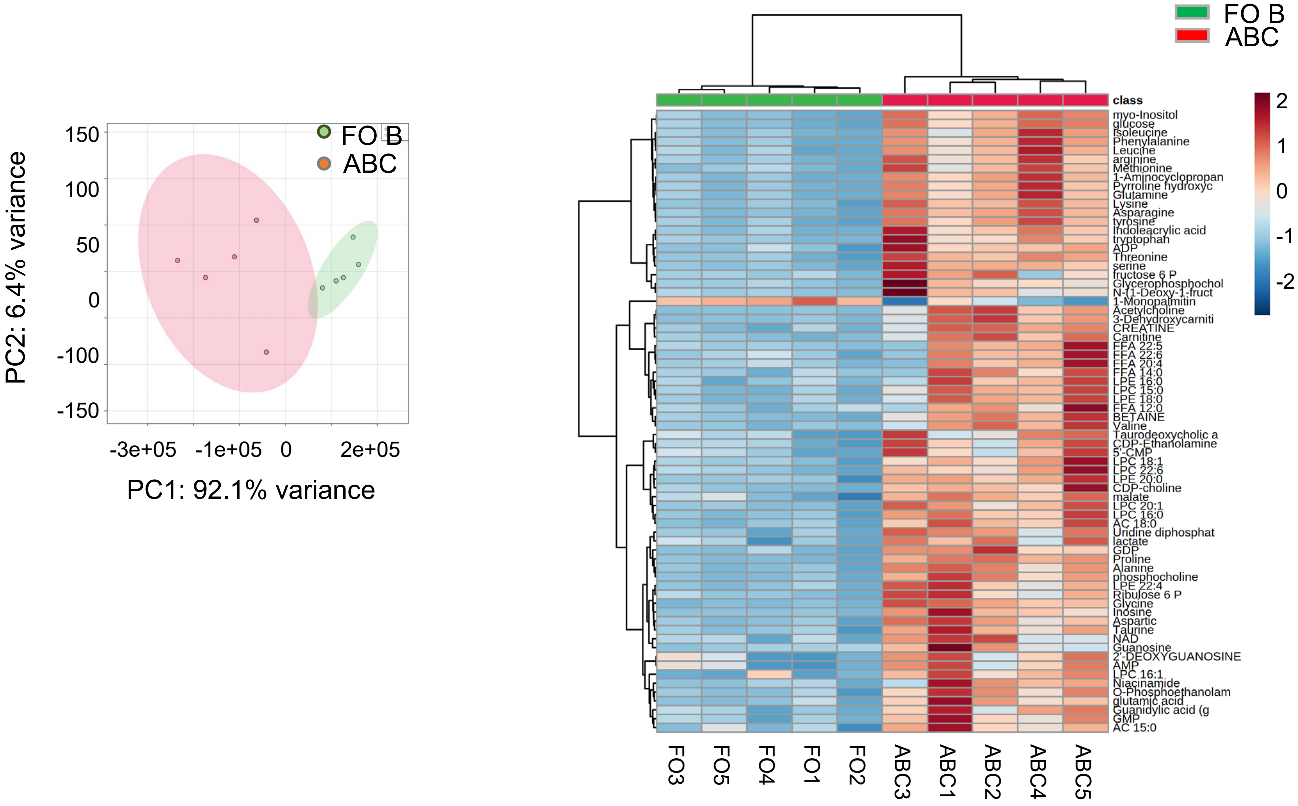

Supplementary figure 6: Metabolomic analysis of FO B cells and ABCs. Principal component analysis of metabolites from B cell subsets (green- FO B cells and pink- ABCs) (on the left-hand side). The heat map of differential metabolites from the B cells (on the right-hand side). Colors indicate Z score with red representing increased and blue representing decreased with respect to control. Each column represents an individual mouse (as written on the bottom of the map).

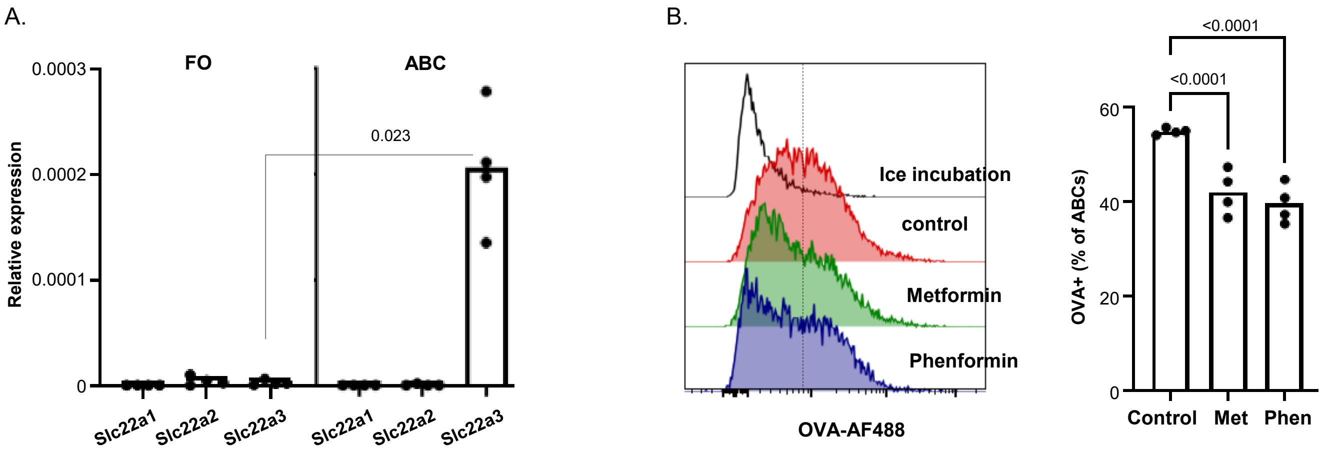

Supplementary figure 7: Metformin receptor expression in B cells and suppression of OVA uptake by metformin and phenformin. (A) Metformin receptors, *Slc22a1*, *Slc22a2* and *Slc22a3*, were measured from isolated ABCs or FO B cells by qRT-PCR. (B) ABCs were exposed to medium alone (control), metformin or phenformin for 2 hours and 10 µg/ml of OVA-AF488 was incubated for 30 min. OVA-positive ABCs were analyzed by flow cytometry and percent positive was calculated based on the ice incubation control (grey line on top histogram). Each dot represents an individual mouse, and the bar represents the mean. (n=2). Unpaired Mann-Whitney was used for statistical test of (A), and one-way ANOVA was used for (B), and Sidak's correction was applied for multiple comparisons.
